# Rare osteodysplasia of the temporal bone

**DOI:** 10.1016/S1808-8694(15)31315-X

**Published:** 2015-10-20

**Authors:** Marcos L. Antunes, José R.G. Testa, Ricardo Frazatto, José A.F. Barberi, Rogério F.N.D. Silva

**Affiliations:** 1 Master, Ph.D. studies under course. Discipline of Otorhinolaryngology – Unifesp; 2 Faculty, Discipline of Pediatric Otorhinolaryngology. Federal University of Sao Paulo – Unifesp; 3 Master in Otorhinolaryngology and Head and Neck Surgery. Discipline of Otorhinolaryngology – Unifesp; 4 Resident Physician. Discipline of Otorhinolaryngology – Unifesp

**Keywords:** osteopetrosis, osteodysplasia, temporal bone

## Abstract

Temporal bone osteodysplasia can produce many different symptoms, such as involvement restricted to the temporal bone or impairment of other bones. We consider, in this study two entities that are rare osteodysplasia cases, which are osteopetrosis and Camurati-Engelmann disease, the latter being extremely rare. We present two cases of benign form of osteopetrosis (Albers-Schulenburg's disease), a patient of 11 years old and another one of 48 years old, both male, and a patient of 28 years old, female, with Camurati-Engelmann's disease. The facial palsy was a manifestation in two of the patients. We discuss some aspects about the clinical manifestations, radiological findings, as well as differential diagnostic and therapy in view of the complications of the diseases.

## INTRODUCTION

Osteodysplasia are characterized by affections to bone density, which may exclusively affect the head or other bones. Osteodysplasia that affects the temporal bone can also be limited to it, such as otospongiosis or systemic abnormalities such as the majority of fibrous dysplasia^1^ and Paget disease. Otospongiosis is the most common one^1^. Rare pathologies with temporal bone affections are also described in the literature, with significant manifestations such as rapidly progressive hearing loss, dizziness and peripheral facial palsy.

The authors described 3 patients with rare temporal bone osteodysplasia, two affected by osteopetrosis, and one patient had a rare disease named Camurati-Engelmann disease, showing different aspects that may support diagnosis, in addition to clinical management in view of clinical implications.

Osteopetrosis is a rare disease (up to 1970, there were only about 350 reports in the whole literature^2^); it has unknown etiology, progressive, hereditary, characterized by increase in bone density of skull base and scalp bone resulting from reduced bone reabsorption by osteoclast dysfunction detected on the endochondral and periosteal layers^3^, whereas the function of osteoblast was normal, occurring strictly with cranial nerves foramens and secondary neurological symptoms. The most affected cranial nerves are: optical, trigeminal, vestibular-cochlear and facial, the latter characterized by acute recurrent episodes similar to Bell's palsy, but with worsening of muscle weakness in each episodes^2^, ^4^. Bones were hard owing to the higher amount of calcium salts, but they did not present organization to stress, showing brittleness^2^, ^4^, ^5^. Diagnosis is radiological, showing exaggerated increase of bone with reduction of medullary cavity and diploic space^2^, ^5^. This radiological aspect is important to differentiate osteopetrosis from other pathologies, especially in children with peripheral facial palsy, from what can be the first sign of the disease^4^, or in children or adults with recurrent episodes.

There are two clinical forms of this entity^2^, the malignant, autosomal recessive form, with worse prognosis that affects more children than adults, characterized by obliteration of bone marrow for abnormal bone tissue^4^, ^6^, with anemia and neutropenia (which are the main causes of death in these children), extramedullary hematopoiesis, liver and spleen increase, optical atrophy by direct compression of nerve or resulting from vascular affection^9^, facial palsy, severe sensorineural hearing loss, mental retardation, hydrocephalus, multiple fractures and hypocalcemia. Histopathological studies have noticed dehiscence of facial nerve with herniation to the niche of oval window and absence of inner ear affection^7^. This form is fatal up to approximately the 2nd decade of life. The benign form is autosomal dominant (Albers-Schöenberg disease), with better prognosis, affecting adolescent and adults, and it may be asymptomatic or characterized by macrocephaly, mandible widening, proptosis (because of orbital bone affection), conductive hearing loss (involvement of middle ear structures), there may be sensorineural hearing loss by bone constriction of cochlear nerve at internal acoustic meatus (MAI)^3^, normal intelligence, rare involvement of optical nerve. Syndactilia or nail malformations may be helpful for diagnosis. The benign form presents two types^6^, ^8^, ^9^: I) marked sclerosis of scalp bone, narrowing of auditory tube, internal and external acoustic meatus (sensorineural loss), conductive hearing loss by increase in rigidity of ossicle chain and obliteration of round and oval windows^8^; it affects mainly trigeminal and vestibular cochlear nerves, but it may also affect facial nerve, marked sclerosis on the skull base, without MAI narrowing, with involvement of facial nerve (facial paralysis in 100% of the cases), and it may also affect vestibular-cochlear nerve.

There is no treatment to prevent progression of the disease, but decompression of cranial nerves 2, 7 and 8 has been known to prevent functional losses from bone compression. Facial nerve decompression in mastoid and tympanic segments has already been conducted, but some authors^9^ believe it does not prevent recurrence of acute facial palsy or does not completely improve cases with compression by narrowing of internal acoustic canal, requiring complete decompression of the nerve^2^ (via mastoid or middle fossa).

Camurati-Engelmann disease is a rare autosomal dominant hereditary disease of variable penetrance, reported by Camurati in 1922 and later characterized by Engelmann in 1929^10^, involving diaphysis of long bones that may affect the skull base, clavicle, and cause neuromuscular abnormalities. The progression of bone sclerosis may also affect cranial nerve foramens, causing deficits such as facial palsy, hearing and visual loss, vascular disorders and facial hypoesthesia. Hearing loss occurs in 18% of the cases^10^, and it may be conductive, by fixation of stapes to the oval window, mixed or sensorineural loss by stenosis of internal acoustic canal. Radiological findings of the skull include skull base necrosis and frontal and occipital bone necrosis, and there may be impairment of mandible, maxilla and zygoma^10^.

Treatment may be surgical through decompression of affected nerves and it may require approach of internal acoustic canal or rehabilitation with conventional hearing aids or cochlear implant^10^ (relative to hearing loss sequelea), depending fundamentally on cochlear reserve.

## CASE REPORT

### *Case 1*

D.S., male, 11 years, Black descendent. Hearing loss on the left, low height, reduction of visual acuity. Widening of forehead, hyperchromic maculla of jugal mucosa and skin, syndactilia of fingers and partially of toes, varus tights, hypertherolism and telecanthus.

Audiometric assessment: moderate conductive hearing loss on the left.

Skull x-ray: diffuse sclerosis.

Temporal bone CT scan: head bone hyperdensity.

Clinical evolution: The patient was stable concerning hearing level (5 years after diagnosis), but had low height considering the treatment in course directed to reducing bone growth.

### *Case 2*

J.A.J., male, 48 years, Caucasian. Anacusis on the left, peripheral facial palsy on the left and rotation dizziness. Later, anacusis on the right ear. Widening of generalized skull.

Vestibular examination: Bilateral Deficit Peripheral Vestibular Syndrome.

Audiometric assessment: bilateral anacusis, type A tympanometric curve, absence of bilateral reflexes.

Head CT scan: generalized hypertrophic osteopathy with spared ethmoid.

Temporal bone CT scan: hyperostotic craniodysplasia with obliteration of internal acoustic canal.

ABR: absence of bilateral responses.

Clinical evolution: The patient preferred clinical treatment and died 3 years after onset of facial palsy of intracranial hypertension (owing to increase in head bone tissue).

### *Case 3*

C.L.G.S., female, 28 years, Caucasian.

Facial palsy on the right for 2 months with no improvement, with insidious onset hearing loss, progressive worsening and bilateral tinnitus. After 1 month of facial palsy on the right, facial palsy on the left was detected. She was followed up by Orthopedics and Genetics with diagnostic hypothesis of Camurati-Engelmann disease.

ENT examination: Peripheral facial palsy GIII on the left and GV on the right (House-Brackmann).

Hilger: 1.0mA on the left and non-excitable on the right.

Pure tone audiometry, vocal discrimination and immittanciometry: bilateral moderate to severe mixed loss, 10dB to 20dB gap, descending curve. Absence of stapedial reflexes, type A tympanometry.

ABR: delay in latencies I, III and IV bilaterally, more marked on the right.

Electroneuromyography: bilateral dennervation without signs of reinnervation.

Temporal bone CT scan: osteoblastic affections of skull base in addition to narrowing of both internal acoustic canals.

Clinical evolution: Indicated surgery for total decompression of facial nerve on the right, patient preferred clinical treatment and maintained the same grade of facial palsy.

**Photo 1:**
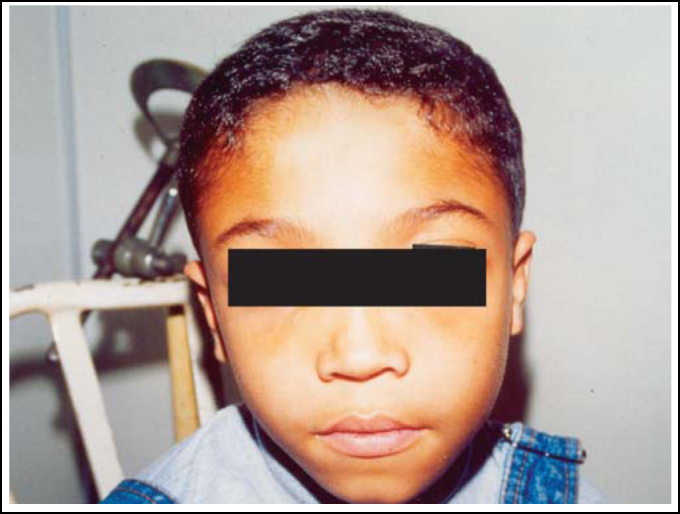
Case 1: patient aged 11 years, male, with benign osteopetrosis. Observe widening of forehead and hypertherolism.

**Photo 2:**
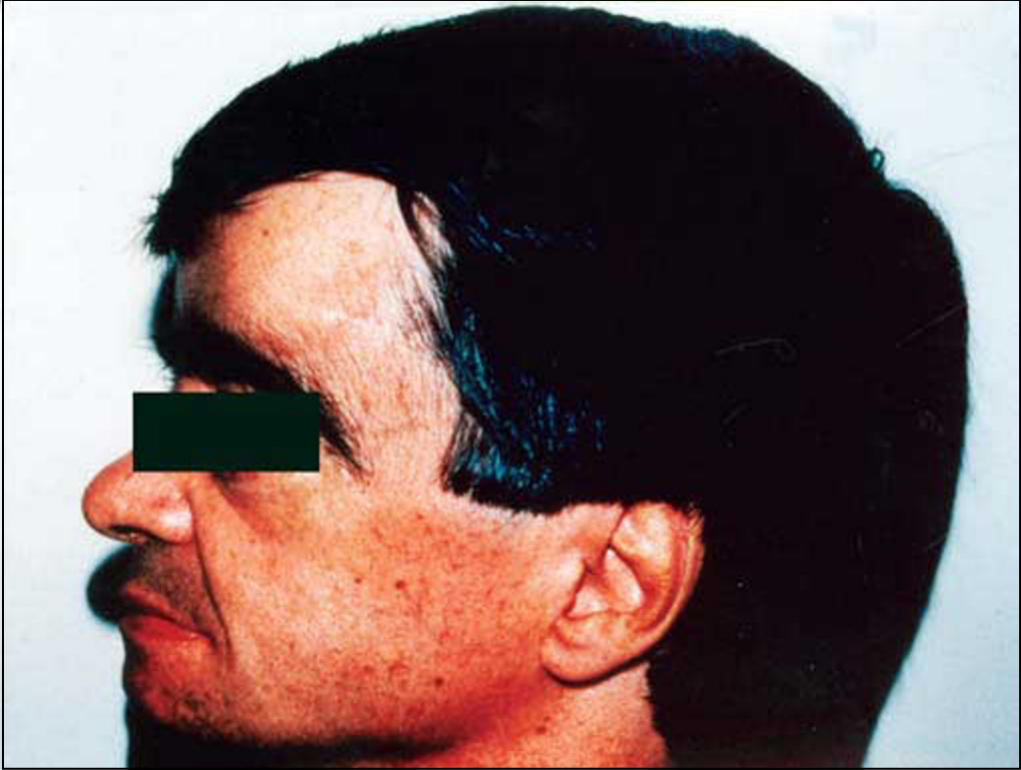
Case 2: patient aged 48 years, male, with progressive osteopetrosis. Note anterior-posterior widening of skull.

**Photo 3:**
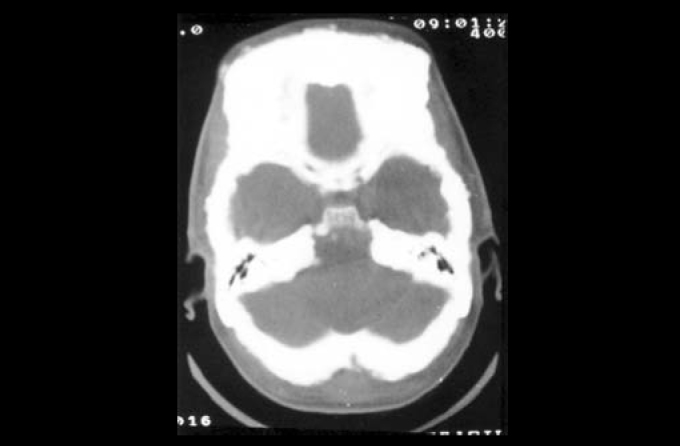
Case 2: CT scan (axial section) showing hyperostotic craniodysplasia, especially in the frontal region, but with diffuse affection.

**Photo 4:**
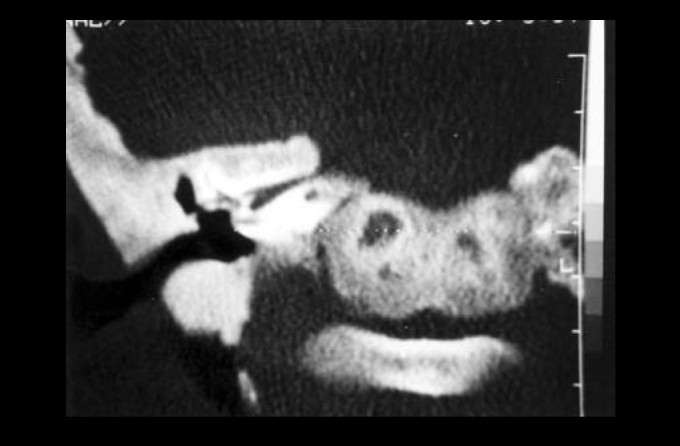
Case 3: Temporal bone CT scan (coronal section) in patient with Camurati-Engelmann disease, evidencing impairment of temporal bone, bone compression of internal acoustic canal on the right.

**Photo 5:**
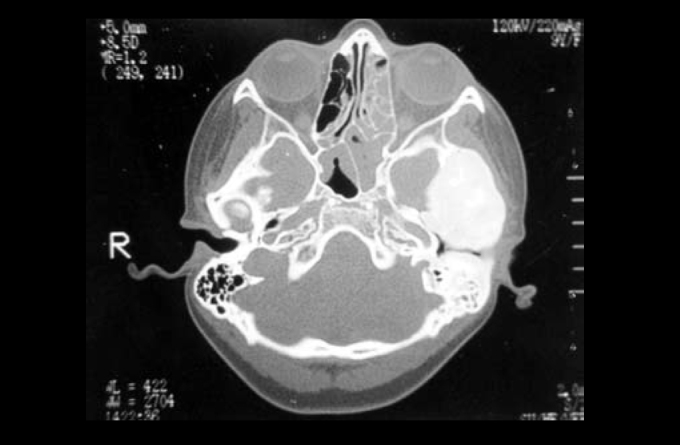
Temporal bone CT scan (axial section) of a patient with fibrous dysplasia of temporal bone on the left, as differential diagnosis. Note the spongiotic bone.

## DISCUSSION

Both cases of osteopetrosis presented are benign forms of the disease, that is, it affects adolescents and adults. We can see that presentation is variable. In patient number 1, main symptoms were reduction of visual acuity and hearing loss, represented by conductive loss. The 2nd patient presented anacusis, peripheral facial palsy and vertigo, characterizing cranial nerve compression, which was confirmed by CT scan by narrowing of internal acoustic canal. Physical examination can support us in the diagnosis, such as presence of widening of head or forehead, syndactilia^8^ and affection to other bones. However, the most helpful exam is CT scan, especially temporal bone scan, because we can check the diameter of internal acoustic canal in addition to impairment of facial nerve canal. The presence of exaggerated increase of bone with rarefaction of medullary cavity supports diagnosis^2^. In the patient affected by Camurati-Engelmann disease, initial symptom related to temporal bone affection was slowly progressive hearing loss. What attracted our attention and made the patient come for medical care was facial palsy, which evolved to bilateral presentation within one month, considering that there was delay between the first manifestations of facial nerve involvement and the first visit (2 months), which worsens prognosis when it comes to compression the nerve or narrowing of canal. The first manifestations of the disease are normally in long bones, but we should be attentive to the possibility of having temporal bone affection so that treatment can be started as early as possible.

We should make the differential diagnosis with other cranial-facial affections resulting from osteodysplasias such as Paget disease, fibrous dysplasia, especially in its pagetoid or sclerotic forms (but in dysplasia there is expansion to medullary cavity), osteogenesis imperfecta (Van Der Hoeve syndrome)^9^, exostosis and exuberant osteoma, among others.

As to hearing loss of these patients, the use of hearing aids should be considered when the patients does not have anacusis. When the patient has bilateral anacusis, we can consider cochlear implant, without ignoring the fact that cochlear nerve may be compressed when there is internal acoustic canal stenosis, which may lead to failure in treatment with implant, but indirect signs of feasibility of nerve should be taken into consideration, such as absence of facial palsy or vestibular symptoms^10^. Auditory brainstem response audiometry may be useful to support therapeutic decision. In our adult patient with osteopetrosis, ABR showed absence of bilateral response, which theoretically would prevent cochlear implant. The same patient died years after owing to excessive bone growth and compression of vital structures.

Impairment of facial nerve is relatively common in forms of the disease that affect the temporal bone and, in the description of our three cases, two presented facial palsy (both with canal impairment confirmed by CT scan). The paralysis may indicate evolution of the disease in a stage of further involvement of the bone, and it may happen before the onset of sensorineural loss (as observed in our patient with Camurati-Engelmann disease).

Facial nerve decompression should be always considered when paralysis evolution is unfavorable (owing to grade of paralysis and electrical tests of prognosis), and we should bear in mind that total decompression of the nerve is preferable owing to MAI impairment and also because facial palsy may be recurrent and, in these cases, it may have an evolution that is less favorable.

## CLOSING REMARKS

Temporal bone osteodysplasias are rare diseases, except for otospongiosis, with bone neoformation that may manifest through the temporal bone or involve other bones in the skull or even extracranially. Clinical manifestations of rare osteodysplasias such as osteopetrosis and Camurati-Engelmann disease depend on the site of involvement, which may present with conductive, mixed or sensorineural hearing loss or even anacusis, vertigo, facial palsy, reduction of visual acuity, among other symptoms. We should bear in mind the possible differential diagnoses and the need in some cases to have surgical intervention such as facial nerve decompression or cochlear implant in bilateral profound hearing losses.

## References

[1] Cruz OLM, Pessoto J, Pezato R, Alvarenga EL. (2002). Osteodistrofias do osso temporal: Revisão dos conceitos atuais manifestaçõ es clínicas e opçõ es terapêuticas.. Ver Bras Otorrinolaringol.

[2] Hamersma H. (1974). Total decompression of the Facial Nerve in Osteopetrosis (Marble Bone Disease - Morbus Albers – Schönberg).. ORL J Otorhinolaryngol Relat Spec.

[3] Suga F, Lindsay JR. (1976). Temporal bone histopathology of osteopetrosis.. Ann Otol Rhinol Laryngol.

[4] Myamoto RT, House WF, Brackmann DE. (1980). Neurotologic manifestations of the osteopetrosis.. Arch Otolaryngol.

[5] Myers EN, Stool S. (1969). The temporal bone in osteopetrosis.. Arch Otolaryngol.

[6] Milroy CM, Michaelis L. (1990). Temporal Bone Pathology of Adult – Type Osteopetrosis.. Arch Otolaryngol Head Neck Surg.

[7] Yarington CT, Sprinkle PM. (1967). Facial palsy in osteopetrosis.. Jama.

[8] Bollerslev J, Grontved A, Anderson PE. (1988). Autossomal dominant type osteopetrosis: an otoneurological investigation of two radiological types.. Laryngoscope.

[9] Hamersma H. (1970). Osteopetrosis (Marble Bone Disease) of the temporal bone.. Laryngoscope.

[10] Friedland DR, Wackym PA, Rhee JS, Finn MS. (2000). Cochlear implantation for auditory rehabilitation in Camurati-Engelmann disease.. Ann Otol Rhinol Laryngol.

